# Absolute blood volume and long-term survival in chronic hemodialysis patients

**DOI:** 10.1177/03913988241296405

**Published:** 2024-11-07

**Authors:** Joachim Kron, Stefanie Broszeit, Til Leimbach, Susanne Kron

**Affiliations:** 1 KfH Kidney Center Berlin-Köpenick, Berlin, Germany; 2 Department of Nephrology, DRK Clinics Berlin-Köpenick, Berlin, Germany

**Keywords:** Chronic hemodialysis, absolute blood volume, long-term survival, Kaplan–Meier analysis, impaired left ventricular ejection fraction

## Abstract

Current online hemodiafiltration devices can be used to determine the absolute blood volume in clinical practice using the dialysate bolus method. Most of publications on this method have focused on preventing intradialytic complications. The influence of absolute blood volume on long-term prognosis has not been reported yet. A total of 79 participants in a previous study about absolute blood volume were followed for 5 years. Patients with a specific blood volume above (*n* = 45) and below 75 ml/kg (*n* = 34) respectively were compared with regard to survival using Kaplan–Meier analysis. Patients with a specific blood volume below 75 ml/kg had a significantly higher overall 5-year survival rate than patients above 75 ml/kg (70% vs 39%, *p* = 0.0233). In patients without cardiac dysfunction, there were no significant differences in 5-year survival between a specific blood volume below or above 75 ml/kg (66% vs 51%). A specific blood volume above 75 ml/kg was associated with an increased mortality in patients with mildly impaired left-ventricular systolic ejection fraction of 40%–59%, whereas in patients with normal blood volume this cardiac impairment did not impact mortality (22% vs 90% 5-year survival, *p* = 0.0036). This demonstrates the significance of optimum volume control for long-term survival particularly in cases of reduced cardiac function.

## Introduction

The expansion of intravascular volume causes long-term cardiovascular damages whereas intravascular volume depletion contributes to intradialytic hypotension. Therefore, absolute blood volume (BV) is the central issue of volume management in hemodialysis therapy with the aim is to keep blood volume within a physiological range as far as possible. Ten years ago, we developed the dialysate bolus method to determine absolute blood volume in clinical practice with current on-line hemodiafiltration machines equipped with a blood volume monitor and an online bolus function. Absolute blood volume is calculated from the increase in relative blood volume after an infusion of a well-defined volume bolus of ultra-pure dialysate into the extracorporeal circulation.^1^ Since then, 26 studies have been published using this method.

However, the influence of absolute blood volume on long-term survival of hemodialysis patients has not been reported to date. Although it has long been known that an overload in extracellular volume (ECV) is associated with an increased mortality.^2–4^ Evidence of randomized controlled trials that bioimpedance-based interventions reduce the mortality is lacking.^5–6^ The aim of this study was to investigate the influence of the intravascular volume status on survival in hemodialysis patients.

## Methods

All participants (*n* = 79) in a previous study about absolute blood volume^7^ were followed for 5 years. Patients provided written informed consent as approved by the Ethics Committee of the Charité Berlin (approval number EA1/036/17).

Patients with severe volume expansion (over 4 L), clinical symptoms of heart failure (NYHA class >II), cardiac dysfunction with left-ventricular ejection fraction (LVEF) <40% on echocardiography, or metallic implants were excluded from that study. Only stable patients with a well-functioning fistula without shunt recirculation were included at that time. Shunt recirculation was measured using the blood temperature monitor (BTM), which is an integral part of the dialysis machine.

The study was performed with the 5008 on-line hemodiafiltration machine (Fresenius Medical Care (FMC), Bad Homburg, Germany) equipped with a blood volume monitor to record relative blood volumes. Absolute blood volume was calculated from the increase in relative blood volume after an infusion of a 240 mL dialysate bolus. Theory and method are described elsewhere in more detail.^1,7–9^

Before treatment, extracellular volume, and volume overload (VO) were evaluated by bioimpedance spectroscopy (BIS) using the body composition monitor (BCM, Fresenius Medical Care (FMC), Bad Homburg, Germany).

### Statistics

Survival analysis was performed with the Kaplan–Meier estimation and the log-rank test.

Normally distributed data assessed by Shapiro–Wilk test are provided as mean ± standard deviation (SD). Significance of differences was assessed by *t*-test, Mann–Whitney *U* test, chi-square test, or Fisher’s exact test. The association between variables was examined by the Pearson correlation coefficient (*r*). A probability *p* < 0.05 was assumed as significant to reject the null-hypothesis.

## Results

Of the 79 patients included in that study, 40 remained on hemodialysis treatment after 5 years, 31 patients died, 7 underwent transplantation, and 1 patient changed the dialysis facility. Patient characteristics are given in Table 1.

**Table 1.** Characteristics of 79 patients followed for over 5 years with a specific blood volume above or below 75 ml/kg.

	All patients (*n* = 79)	Below 75 ml/kg (*n* = 34)	Above 75 ml/kg (*n* = 45) *p*
Age (years) 0.679	69.0 ± 14.8	69.8 ± 14.5	68.4 ± 15.1
Gender (f) 0.721	32 (40%)	13 (38%)	19 (42%)
Diabetes 0.200	17 (21%)	5 (15%)	12 (27%)
LVEF 40%–59% 0.332	19 (24%)	10 (29%)	9 (20%)
Dialysis vintage (months) 0.116	98 (7–414)	82 (21–414)	108 (7–356)
Specific blood volume (ml/kg) <0.001	77.3 ± 9.6	70.3 ± 5.3	82.6 ± 8.7
Absolute blood volume (l) 0.269	5.76 ± 1.54	5.54 ± 1.13	5.93 ± 1.79
Extracellular volume (l) 0.591	17.91 ± 3.90	18.17 ± 3.25	17.69 ± 4.34
Blood volume to 0.001	0.321 ± 0.0390	0.3052 ± 0.0345	0.3325 ± 0.0376
Volume overload (l) 0.004	1.85 ± 1.22	1.41 ± 1.07	2.13 ± 1.06
Volume overload in % ECV <0.001	10.26 ± 5.84	7.58 ± 5.61	12.29 ± 5.20
Dry weight (kg) 0.057	74.8 ± 19.2	79.5 ± 19.9	71.2 ± 18.1
Body mass index 0.049	27.25 ± 5.53	28.65 ± 6.53	26.18 ± 4.42
Fat tissue index 0.059	13.62 ± 6.07	15.10 ± 7.33	12.50 ± 4.69
Lean tissue index 0.820	12.67 ± 3.08	12.78 ± 3.37	12.59 ± 2.88

LVEF, left ventricular ejection fraction; ECV, extracellular volume; p, specific blood volume below versus above 75 ml/kg.

Between euvolemic patients (specific BV <75 ml/kg, *n* = 34) and patients with a specific BV >75 ml/kg (*n* = 45) there were significant differences in blood volume, volume overload, and blood volume to ECV ratio. Euvolemic patients had a higher body mass index (BMI) and tended to have a higher fat tissue index. The correlation between BV and BMI was significant (*p* < 0.001) and similar in both groups (*r* = 0.69 and 0.74).

**Figure 1.**
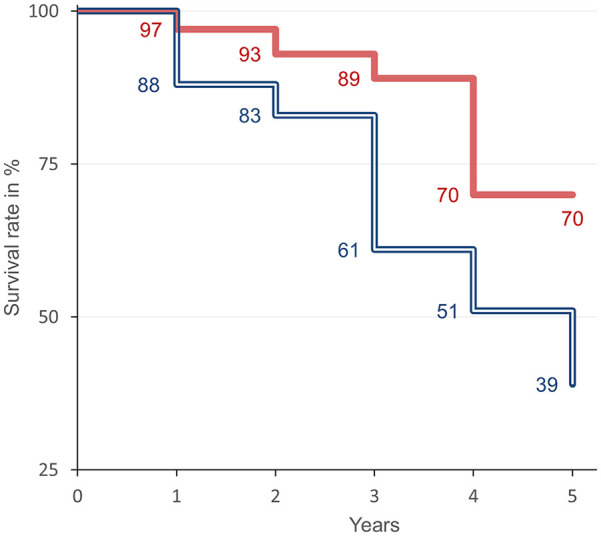
Kaplan–Meier analysis in patient with absolute blood volume below (full line, red) and above (doubled line, blue) 75 ml/kg. The all-cause mortality in a follow-up period of 5 years was significantly different (log-rank test *p* = 0.0233).

Patients with specific BV <75 ml/kg had a significantly higher 5-year survival rate of 70% (log-rank test *p* = 0.0233) compared to 39% in patients with specific BV >75 ml/kg (Figure 1).

**Figure 2.**
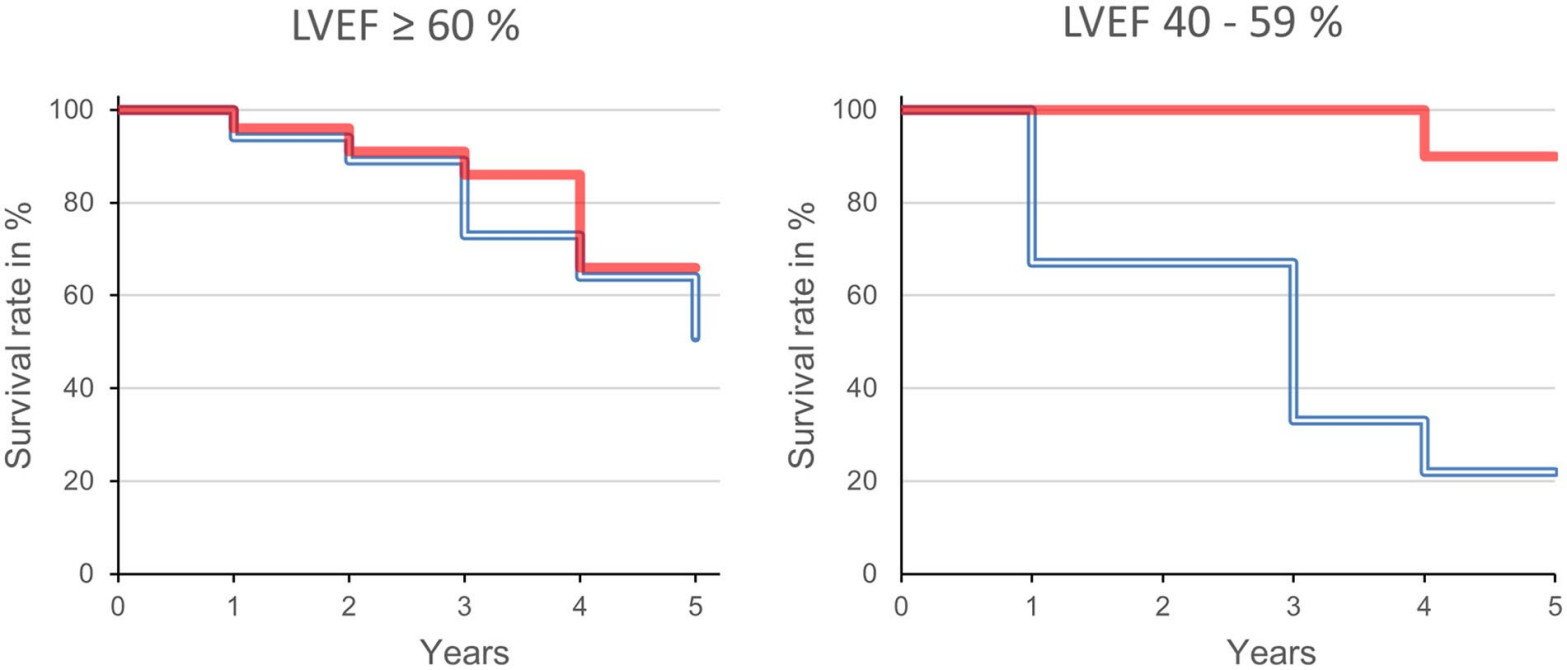
Kaplan–Meier analysis in patient with normal (left) and mildly reduced left ventricular ejection fraction (LVEF) of 40%–59% (right). Absolute blood volume below (full line, red) and above (doubled line, blue) 75 ml/kg. The differences in patients with impaired cardiac function are significant (log-rank test *p* = 0.0036).

While there were no significant differences in 5-year survival between patients with specific BV below or above 75 ml/kg without cardiac dysfunction (66% vs 51%, *p* = 0.354), the 5-year survival rates differed significantly in patients with impaired LVEF (*n* = 19; Figure 2). Impaired LVEF had no impact on mortality in patients with a specific BV below 75 ml/kg (90% 5-year survival), whereas only 22% of patients with specific BV above 75 ml/kg survived for 5 years (*p* = 0.0036).

## Discussion

This is the first report on the prognostic impact of intravascular volume status in chronic hemodialysis patients. Despite the exclusion of patients with significant volume overload, clinical signs of heart failure and reduced LVEF <40%, differences are already visible in patients with moderately increased blood volume and very mildly impaired LVEF. This results in the following new insights:

Firstly, there are no significant differences in 5-year survival of patients with moderately increased and normal BV without impaired cardiac function (Figure 2).
Secondly, a moderately increased BV is already a risk factor for mortality in cases of mild LVEF impairment.
Thirdly, mild cardiac dysfunction does not increase mortality in patients with normal BV.

The latter is of considerable importance in clinical practice. If hemodialysis patients with cardiac dysfunction are maintained in a euvolemic status, their mortality is not increased. Surprisingly, these patients had the best 5-year survival rates in our study. We don’t have a conclusive explanation for this. May be that knowledge of cardiac risk has resulted in reduced fluid intake in some patients. Nevertheless, this demonstrates the importance of strict volume control, particularly in cases of reduced cardiac function. We should therefore inform our patients in detail about these quite optimistic findings. Our findings might confirm the integration of absolute blood volume measurement in volume management strategies.^10^

BV related to kg body mass (specific BV) is underestimated in patients with elevated fat mass.^11–13^ Our patients in the group with a specific BV below 75 ml/kg had an approximately 8 kg higher body mass, a significantly higher BMI, and a slightly higher fat tissue index (Table 1). In some of these patients, the specific blood volume may have been underestimated, and therefore, they have been classified in the group with normal BV. However, there is no bias because these patients were in the group with the lower mortality. Moreover, the correlation between BV and BMI was significant and similar in both groups. This is in contrast to a study by Schmiedecker et al. concerning the relationship between BV and BMI.^12^

It is a limitation of this study that the results are from the follow-up of patients from a clinical study with a different objective^7^ but not from a prospective survival study. Furthermore, the examined patients are not fully representative of the overall dialysis population.

This work is a step toward improved patients’ outcome and quality of treatments, especially when the method of absolute BV measurement and ultrafiltration control would be implemented into the dialysis devices. A prescribed target of absolute BV can technically be reached at the end of each dialysis.^14–16^

In conclusion, this is the first study to highlight the importance of absolute BV for long-term survival in chronic hemodialysis patients. The results demonstrate the significance of strict volume control, particularly in cases of reduced cardiac function. Nevertheless, the conclusions are preliminary and need to be confirmed in well-designed prospective long-term studies including high-risk patients.
